# Edith Thomas’s Blindness

**Published:** 1891-03

**Authors:** 


### EDITH THOMAS'S BLINDNESS.

From a report of the Superintendent of the Perkins Institute for the Blind, we learn that until recently Edith Thomas has seemed unconscious of her blindness, and although it was freely spoken of in her presence, while her hearing still remained she has from the beginning insisted that she could see. Before she was able to leave her bed her mother went down and asked her child if she could see her. Edith stretching out her little hands and passing them over her mother's head, answered in happy tones of childhood. "Course I can see you !" And a little later, when she began to run about the house, and the children remarked that she was blind, she indignantly denied it, insisted that she could see every thing, and appealed to her mother, saying, "I aint blind, is I, mamma ?" She would put on some favorable article of dress, which she had worn before she lost her sight, go to the mirror, and turning from side to side, survey herself with childish delight. Thenceforward she continued this apparent use of the eyes with seemingly the same degree of satisfaction as if physical vision remained. If she had a new hair ribbon she would go to the glass to try it on and would even hold a hand glass, looking into it all the while her hair was being combed.

